# Insights on the DNA Stability in Aqueous Solutions of Ionic Liquids

**DOI:** 10.3389/fbioe.2020.547857

**Published:** 2020-10-14

**Authors:** Teresa B. V. Dinis, Fani Sousa, Mara G. Freire

**Affiliations:** ^1^Department of Chemistry, CICECO – Aveiro Institute of Materials, University of Aveiro, Aveiro, Portugal; ^2^CICS-UBI – Health Sciences Research Center, Universidade da Beira Interior, Covilhã, Portugal

**Keywords:** DNA, interactions, ionic liquids, native conformation, nucleic acid, stability

## Abstract

Deoxyribonucleic acid (DNA) carries the genetic information essential for the growth and functioning of living organisms, playing a significant role in life sciences research. However, the long-term storage and preservation of DNA, while ensuring its bioactivity, are still current challenges to overcome. In this work, aqueous solutions of ionic liquids (ILs) were investigated as potential preservation media for double stranded (dsDNA). A screening of several ILs, by combining the cholinium, tetrabutylammonium, tetrabutylphosphonium, and 1-ethyl-3-methylimidazolium, cations with the anions bromide, chloride, dihydrogen phosphate, acetate, and glycolate, was carried out in order to gather fundamental knowledge on the molecular features of ILs that improve the dsDNA stability. Different IL concentrations and the pH effect were also addressed. Circular dichroism (CD) spectroscopy was used to evaluate the conformational structure and stability of dsDNA. IL-DNA interactions were appraised by UV-Vis absorption spectrophotometry and ^31^P nuclear magnetic resonance (NMR) spectroscopy. The results obtained demonstrate that pH has a significant effect towards the dsDNA stability. Amongst the ILs investigated, cholinium-based ILs are the most promising class of ILs to preserve the dsDNA structure, in which electrostatic interactions between the cholinium cation and the DNA phosphate groups play a significant role as demonstrated by the ^31^P NMR data, being more relevant at higher IL concentrations. On the other hand, the denaturation of dsDNA mainly occurs with ILs composed of more hydrophobic cations and able to establish dispersive interactions with the nucleobases environment. Furthermore, the IL anion has a weaker impact when compared to the IL cation effect to interact with DNA molecules. The experimental data of this work provide relevant fundamental knowledge for the application of ILs in the preservation of nucleic acids, being of high relevance in the biotechnology field.

## Introduction

Deoxyribonucleic acid (DNA) is one of the most important macromolecules in cells, carrying the genetic information essential for the growth and functioning of living organisms. DNA is arranged in a helical stranded structure, but it can adopt different three-dimensional conformations. Because of this structural polymorphism (Renčiuk et al., 2009), experimentally, DNA can be designed to create specific structures, being a powerful tool in many fields of application, such as in the development of advanced materials (Tateishi-Karimata and Sugimoto, 2014), templated chemical synthesis, nanomachines, and biosensors (Gartner et al., 2004; Yamada et al., 2005; Liu and Liu, 2009). On the other hand, the biological significance of DNA as a genetic information carrier places this biopolymer as a hot topic of research in life sciences.

In the past decades, several studies pointed out DNA as a relevant biopharmaceutical for genetic therapy purposes (Uludag et al., 2019), namely in the development of DNA vaccination, in pluripotent stem cells research, cellular therapy in psychiatric diseases (Lissek, 2017) and to induce the expression of therapeutic transgenes (Yin et al., 2014). However, the therapeutic efficacy and biological activity of DNA mainly depends on its structural stability and integrity (Diamantino et al., 2016). Due to its degradation by nucleobases and chemical instability, DNA is not stable in aqueous solutions at room temperature for long periods (Lindahl and Nyberg, 1972; Sasaki et al., 2007). Furthermore, temperature, ionic strength, pH and solvent type, and concentration are critical factors that lead to DNA destabilization (Lindahl and Nyberg, 1972; Cheng and Pettitt, 1992). Long-term storage and preservation of DNA at room temperature, while ensuring its bioactivity, are therefore important issues, motivating the research on effective and sustainable solvents for DNA preservation.

With the appearance of the first air- and water-stable ionic liquids (ILs), the scientific community focused the research on the finding of alternative applications for these compounds (Plechkova and Seddon, 2008), namely in organic chemistry (Sheldon, 2001; Jain et al., 2005; Zhao et al., 2005; Wong et al., 2006), new materials formulations (Hussey, 1994; Bowlas et al., 1996; Gordon et al., 1998; Endres and El Abedin, 2006), biocatalysis (Ventura et al., 2012) and as improved solvation media for a plethora of solutes and biomolecules (Freire et al., 2012; Padrino et al., 2018; Shamshina and Berton, 2020). Despite the relevant properties of most ILs, such as low flammability, and high thermal, and chemical stabilities (Seddon, 1997), they also display tunable properties, being generally described as “designer solvents” (Freire et al., 2012). For instance, the physicochemical properties of ILs can be adjusted to provide adequate aqueous microenvironments for biological applications, such as in gene delivery (Satpathi et al., 2015; Freyer et al., 2016) and long-term storage and structural preservation of nucleic acids (Vijayaraghavan et al., 2010; Mukesh et al., 2013; Pedro et al., 2018).

Several research groups have studied the interactions between DNA molecules and ILs, most of the times focused on the finding of novel solvents for DNA preservation. For instance, Ding et al. (2010) suggested a mechanism of interaction between IL and DNA mainly dependent on the IL concentration in water: for IL concentrations lower than 1.05 wt%, the IL cation is localized at several angstroms of distance from DNA phosphate strand, while the IL hydrophobic chains are in parallel arrangement to the DNA molecule surface; however, for higher IL concentrations, the IL cationic head group is near to the DNA phosphate strand and the IL hydrocarbon chains are perpendicularly attached to the DNA molecule surface. Chandran et al. (2012) described that the electrostatic interactions between ILs and DNA phosphate groups as well as hydrophobic and polar interactions between ILs and DNA major and minor grooves are responsible for dehydration and high stability of DNA macromolecules. More recently, Sahoo et al. (2018) reported the molecular mechanism of binding between DNA and non-toxic ILs composed of a cholinium cation and amino-acid-derived anions, namely glycine, alanine, and proline. The authors showed that IL anions have a negligible effect on binding to DNA, when compared with the cholinium cation. On the other hand, Satpathi et al. (2015) proposed that the IL guanidinium tris(pentafluoroethyl)trifluorophosphate is not involved in specific interactions with DNA but instead leads to the compaction of the DNA structure from coil-to-globule conformation.

Although relevant manuscripts have been published (Ding et al., 2010; Chandran et al., 2012; Satpathi et al., 2015; Sahoo et al., 2018), the existence of variable discussions on the mechanisms responsible for the interactions occurring between ILs and DNA is also affected by a still limited number of studies regarding the IL chemical structure effects. Furthermore, a more comprehensive description of the importance of water molecules in the DNA native structure and stability is still missing when dealing with hydrated ILs or IL-water mixtures. In this work, we investigated a series of hydrophilic ILs (combining different cations and anions) in aqueous solutions in order to extend the scientific knowledge about the DNA stability and binding phenomenon occurring between IL and DNA in aqueous solutions, aiming at identifying promising ILs and adequate concentrations to be used in formulations and extraction/separation processes. Although neat ILs have been investigated as well (Zhao, 2015), the use of IL aqueous solutions presents several advantages when compared with neat ILs. Aqueous solutions of ILs may improve the solubility of biomolecules (Cláudio et al., 2013, 2015), provide a more amenable environment and reduced viscosity (Passos et al., 2014) to maintain the biological activity and structural stability of bioactive compounds, and also represent more sustainable solvents since water is used. Circular dichroism (CD) spectroscopy was used to evaluate the conformational structure and stability of DNA in presence of different ILs at different concentrations. The pH and buffer concentration effects were also investigated. The binding characteristics and molecular mechanisms of IL-DNA interactions were studied by UV-Vis absorption spectrophotometry and ^31^P nuclear magnetic resonance (NMR) spectroscopy. To the best of our knowledge, this work comprises for the first time a screening of several ILs with different cations and anions combinations, allowing a deeper understanding of the ILs molecular features responsible for the DNA stability and structural conformation in aqueous solutions.

## Materials and Methods

### Materials

**FIGURE 1:**
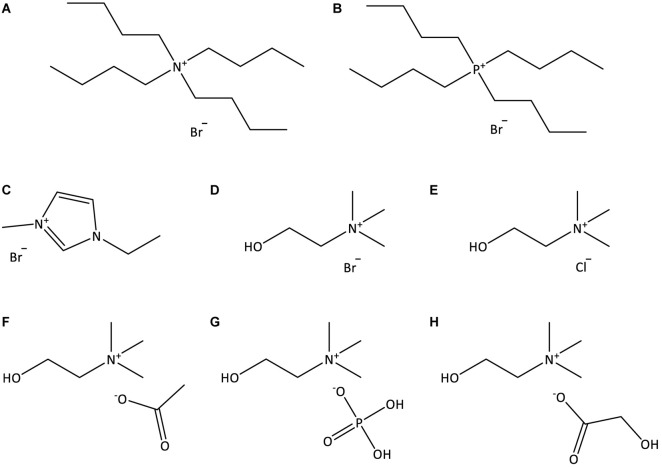
Chemical structures of the ILs used in this work: **(A)** [N_4444_]Br; **(B)** [P_4444_]Br; **(C)** [C_2_C_1_im]Br; **(D)** [N_111(2OH)_]Br; **(E)** [N_111(2OH)_]Cl; **(F)** [N_111(2OH)_][Ac]; **(G)** [N_111(2OH)_][DHP]; **(H)** [N_111(2OH)_][Gly].

Double stranded deoxyribonucleic acid (dsDNA) sodium salt extracted from Salmon testes (CAS no. 9007-49-2), of analytical grade, was purchased from TCI Chemicals. The 260/280 nm absorbance ratio of the DNA stock solution was found to be 1.896, indicating the absence of proteins as contaminants (Saenger, 1984). The ILs studied were: tetrabutylammonium bromide, [N_4444_]Br, tetrabutylphosphonium bromide, [P_4444_]Br, 1-ethyl-3-methylimidazolium bromide, [C_2_C_1_im]Br, (2-hydroxyethyl)-trimethylammonium (cholinium) bromide, [N_111(2OH)_]Br, cholinium chloride, [N_111(2OH)_]Cl, cholinium dihydrogen phosphate, [N_111(2OH)_][DHP], cholinium acetate, [N_111(2OH)_][Ac], and cholinium glycolate, [N_111(2OH)_][Gly]. The molecular structures of the investigated ILs are illustrated in Figure 1. [C_2_C_1_im]Br (99 wt%), [N_111(2OH)_][DHP] (>98 wt%), [N_111(2OH)_][Ac] (>99 wt%), and [P_4444_]Br (95 wt%) were purchased from Iolitec. [N_4444_]Br (98 wt%) was purchased from Fluka. [N_111(2OH)_]Br (>98 wt%) was purchased from TCI chemicals. [N_111(2OH)_]Cl (98 wt%) was provided by Acros Organics. [N_111(2OH)_][Gly] was synthetized by us, by the neutralization of cholinium hydroxide ([N_111(2OH)_]OH) with the respective acid, glycolic acid (1:1.10 mole ratio), at room conditions according to published protocols (Sintra et al., 2015). [N_111(2OH)_]OH (in methanol solution at 45 wt%) was purchased from Sigma-Aldrich. Glycolic acid (99 wt%) was acquired from Acros Organics. Tris (hydroxymethyl) aminomethane (Tris buffer) (>99.8 wt%) was purchased from Pronalab. Hydrochloric acid (HCl) (in water solution at 37 wt%) was from Sigma-Aldrich. Acetone (100 wt%) and ethanol absolute were acquired from Thermo Fisher Scientific. The water used was double distilled, passed by a reverse osmosis system and further treated with a Milli-Q plus 185 water purification apparatus (18.2 MΩ cm at 25°C).

### Experimental Procedure

#### CD Spectroscopy

CD experiments were performed using a Jasco J-1500 CD spectrophotometer. Aqueous solutions containing 1 g dm^–3^ of DNA in 10 mM of Tris–HCl (pH ≈ 7.2) were incubated during 12 h at 25°C with different concentrations of ILs (5, 15, and 30 wt%), and CD spectra were acquired at a constant temperature of 25°C using a scanning speed of 100 nm min^–1^, with a response time of 4 s over wavelengths ranging from 220 to 350 nm. Due to the high interference caused by [C_2_C_1_im]Br in the CD spectrum in the studied wavelengths, for this particular IL, DNA was regenerated using ice cold ethanol (EtOH) in a sample:EtOH ratio of 1:6. The CD spectrum of 10 mM of Tris–HCl (pH ≈ 7.2) was firstly taken as a blank. The recording bandwidth was of 1 nm with a step size of 0.5 nm using a quartz cell with an optical path length of 1 mm. Three scans were averaged *per* spectrum to improve the signal-to-noise ratio. Measurements were performed under a constant nitrogen flow, which was used to purge the ozone generated by the light source of the instrument.

#### UV-Vis Spectrophotometry

The UV-Vis absorption spectra were obtained with a Shimadzu UV-1800, Pharma-Spec Spectrophotometer. To prepare the samples, different weights of ILs were added to a constant concentration of DNA in aqueous solutions, namely 0.03 g dm^–3^ in 10 mM of Tris–HCl (pH ≈ 7.2) (0.21 g dm^–3^ of DNA for the samples composed of [C_2_C_1_im]Br due to the high UV absorption caused by this IL), in order to have IL final concentrations of 5 and 30 wt%. The concentrations of DNA used were chosen to avoid absorbance saturation. Each sample was allowed to stand for equilibration during 12 h at 25°C before the UV absorption spectra were recorded. To remove the background of each IL in UV absorption, solutions of ILs at the same concentrations, yet with no DNA added, were used as standard controls, while 10 mM of Tris–HCl (pH ≈ 7.2) was taken as blank reading. All measurements were performed from 200 to 400 nm and carried out in a quartz cuvette with optical path length of 10 mm.

#### ^31^P NMR

A total of 35 g dm^–3^ of DNA in 10 mM of Tris–HCl (pH ≈ 7.2) aqueous solutions were prepared in different concentrations of ILs (5 and 30 wt%). These solutions were left to stabilize during 12 h at 25°C. NMR spectra of DNA were recorded in a Bruker Avance III operating at 300 MHz, using deuterium oxide (D_2_O) as solvent containing trimethylsilyl propanoic acid (TSP) as the internal reference. The phosphorus chemical shifts of DNA were externally referenced to 5 vol% of orthophosphoric acid.

#### pH Measurements

pH values of the DNA/IL samples were monitored at (25 ± 1)°C using a SevenMulti (METTLER TOLEDO Instruments) with a relative accuracy of ±0.02. All the aforementioned measurements were performed in triplicate.

## Results and Discussion

The evaluation of the dsDNA stability in aqueous solutions of ILs is of high complexity, particularly when aiming the understanding of the interactions occurring between dsDNA and each IL. In the first place, hydration itself has an important impact on the dsDNA structure (McDermott et al., 2017). The helical structure of dsDNA is stabilized by a solvation environment, where changes on hydration can lead to significant changes in the DNA conformation. Furthermore, several hydrophilic ILs composed of distinct ions were investigated in this work, being organized into four classes: cholinium-, [N_111(2*OH*)_]^+^, tetrabutylammonium-, [N_4444_]^+^, tetrabutylphosphonium-, [P_4444_]^+^, and 1-ethyl-3- methylimidazolium-, [C_2_C_1_im]^+^, based ILs. All classes of ILs have bromide as a similar anion. ILs with cholinium as cation were further combined with bromide, chloride, acetate, [Ac]^–^, dihydrogen phosphate, [DHP]^–^, and glycolate, [Gly]^–^, anions.

### dsDNA Conformational Structure in ILs Aqueous Solutions

The dsDNA conformational structure was firstly evaluated by CD assays in order to infer the stability of this macromolecule in aqueous solutions of ILs at different concentrations: 5, 15, and 30 wt%. Apart from the IL concentration and chemical structure, the buffer concentration and pH were evaluated in order to address their impact on the dsDNA stability. It is well-known that the pH influences the dsDNA stability and structural integrity (Lindahl and Nyberg, 1972; Cheng and Pettitt, 1992). In this work, the pH was controlled by the use of Tris–HCl buffer (pH ≈ 7.2) in all solutions, in which different concentrations were also investigated.

Circular dichroism spectroscopic measurements allow to gather information on the DNA secondary structure (Satpathi et al., 2015), being commonly used to monitor nucleic acids structure perturbations (Tateishi-Karimata and Sugimoto, 2014). The dsDNA from salmon testes, used in this work, presents a β-form conformation, exhibiting two characteristic peaks: one positive band at approximately 275–280 nm associated to π–π base stacking and a negative band around 245 nm corresponding to helicity (Williams and Kielland, 1975; Evdokimov et al., 1976).

Double stranded deoxyribonucleic acid aqueous solutions were prepared in Tris–HCl buffered solutions at different concentrations (from 10 to 1000 mM) to initially appraise the buffer concentration effect in the dsDNA structural conformation. Since no significant changes were observed in the DNA secondary structure when increasing the buffer concentration (cf. Supplementary Figure 1), 10 mM of Tris–HCl was selected and used in the following assays.

**FIGURE 2:**
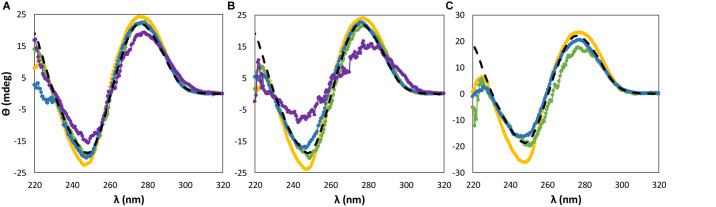
CD spectra regarding the ellipticity, θ, of 0.01 g dm^−3^ of β-DNA (from salmon testes) as a function of wavelength, λ, in 10 mM of Tris–HCl buffer (pH ≈ 7.2) aqueous solutions at different concentrations of bromide-based ILs: **(A)** 5 wt%; **(B)** 15 wt%; **(C)** 30 wt%. (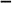) only buffer; (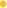) [N_111(2OH)_]Br; (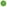) [N_4444_]Br; (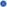) [C_2_C_1_im]Br; (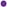) [P_4444_]Br.

**FIGURE 3:**
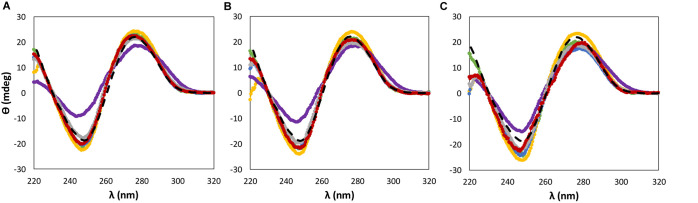
CD spectra regarding the ellipticity, θ, of 0.01 g dm^−3^ of β-DNA (from salmon testes) as a function of wavelength, λ, in 10 mM of Tris–HCl buffer (pH ≈ 7.2) aqueous solutions at different concentrations of cholinium-based ILs: **(A)** 5 wt%; **(B)** 15 wt%; **(C)** 30 wt%. (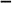) only buffer; (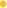) [N_111(2OH)_]Br; (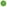) [N_111(2OH)_]Cl; (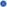) [N_111(2OH)_][Ac]; (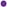) non-buffered [N_111(2OH)_][DHP]; (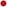) buffered [N_111(2OH)_][DHP]; (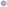) [N_111(2OH)_][Gly].

For almost all ILs and at the three concentrations investigated, dsDNA maintains its β-form conformation with no transition of the native double-helical structure, as shown in Figures 2, 3. Regarding the IL cation effect, shown in Figure 2, and with the exception of [P_4444_]Br, when the remaining ILs are added to DNA aqueous buffered solutions, no significant variations in the CD signal and spectrum of dsDNA are observed. However, with [P_4444_]Br, a significant perturbation in the dsDNA structural conformation is observed at 15 wt% of IL, with a slightly higher effect reflected in the helicity peak than in the base stacking corresponding peak. Furthermore, when using 30 wt% of this IL, no CD spectrum of dsDNA in solution was acquired due to the complete precipitation of DNA.

When dealing with the IL anion effect, being these studies carried out with cholinium-based ILs, less significant changes in the CD signal were observed in the same range of IL concentrations, with the exception of [N_111(2OH)_][DHP] (Figure 3). Aqueous solutions of [N_111(2OH)_][DHP] are highly acidic, being the pH a main factor leading to the destabilization of DNA as demonstrated below.

The effect of the IL concentration on the CD data of nucleic acids has already been reported. Sahoo et al. (2018) reported no conformational changes in both positive and negative bands of β-form DNA along with the increase of [N_1111(2OH)_][Gly] concentration, up to 1.8 wt%. Pabbathi and Samanta (2015) demonstrated no significant modifications on the DNA β-form structure when ranging the *N*-ethyl-*N*-methyl-morpholinium bromide ([Mor1,2]Br) concentration from 6 to 20 wt%. These results are in agreement with the results obtained in this work, at least in the range of IL concentrations studied.

### Effect of pH on the dsDNA Conformational Stability

According to the aforementioned results (Figures 2, 3), both [P_4444_]Br and non-buffered [N_111(2OH)_][DHP] induce significant perturbations in the dsDNA native conformation. Although 10 mM of Tris–HCl (pH ≈ 7.2) was used to keep the pH of all ILs solutions, these two ILs provide highly acidic conditions, with the respective aqueous solutions with pH values ranging between 2 and 4 (cf. Supplementary Table 1 with detailed data). The commercial [P_4444_]Br used contains phosphines as main impurities, whereas [N_111(2OH)_][DHP] provides acidic medium due to the IL anion. It should be noted that the presence of DNA in the aqueous solutions of ILs does not influence the pH values, as experimentally verified. Accordingly, the results obtained in terms of the dsDNA loss of stability seem to be highly affected by the pH and not by the IL chemical structure alone. Some authors already associated acidic properties, along with the increase of the IL concentration, to a significant perturbation of the nucleic acids structure. For instance, Pedro et al. (2018) observed that the structural integrity of ribonucleic acid (RNA) is destabilized in presence of non-buffered [N_111(2OH)_][DHP] that confers acidic conditions to the aqueous medium. However, by the addition of cholinium hydroxide to [N_111(2OH)_][DHP] aqueous solutions to reach a pH *ca.* 7, the authors (Pedro et al., 2018) demonstrated significant improvements in the RNA stability. In the same line, in this work, the stability of dsDNA was further evaluated in buffered [N_111(2OH)_][DHP] by adding cholinium hydroxide up to pH 7. By increasing the pH of the medium there is the improvement of the stability of dsDNA, as shown in Figure 3. These results demonstrate that the IL chemical structure alone is not responsible for the DNA destabilization, but yet the pH plays a significant role.

**FIGURE 4:**
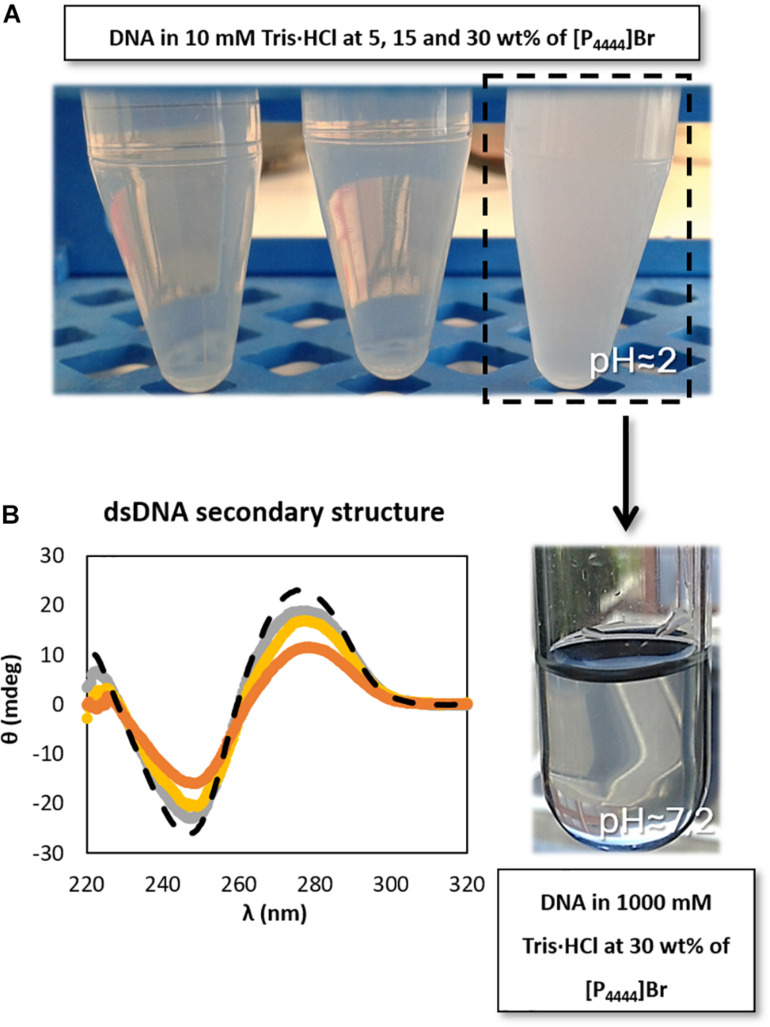
Schematic representation of **(A)** DNA dissolution process with the increase of buffer concentration (increase of pH value) and **(B)** CD spectra regarding the ellipticity, θ, of 0.01 g dm^−3^ of β-DNA (from salmon testes) as a function of wavelength, λ, in 1000 mM Tris–HCl buffer (pH ≈ 7.2). (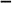) only buffer; (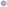) 5 wt% of [P_4444_]Br; (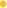) 15 wt% of [P_4444_]Br; (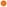) 30 wt% of [P_4444_]Br.

In order to better address the pH effect, a set of studies was additionally performed on the dsDNA stability in aqueous solutions of [P_4444_]Br, since this was the IL that provided the most acidic conditions due to the present impurities (cf. Supplementary Table 1), and where DNA precipitation was observed at 30 wt% of IL. Different concentrations of Tris–HCl buffer were used in 30 wt% [P_4444_]Br aqueous solutions. As shown in Supplementary Figure 2, only with Tris–HCl concentrations from 500 mM it is possible to reach the physiological pH in aqueous solutions containing 30 wt% of [P_4444_]Br. Furthermore, in aqueous solutions composed of [P_4444_]Br at 30 wt% in 1000 mM of Tris–HCl (pH ≈ 7.2) it was observed that the solubility of DNA increases (Figure 4A – the tendency followed by the arrow), thus avoiding the DNA precipitation initially observed, reinforced by the improvement in the maintenance of the dsDNA native conformation (Figure 4B). Our results are in agreement with those published by Vijayaraghavan et al. (2010), who performed a set of experiments to demonstrate the effect of pH on the fluorescence emission intensity of aqueous solutions of DNA. Since fluorescence is the result of the presence of the hydrogen-bonded adenine base in native DNA, its intensity depends on the pH of the medium. In acidic conditions, the authors attributed the increase of the intensity to the increased protonation of adenine (Vijayaraghavan et al., 2010). Overall, and although pH plays a significant role since the protonation of DNA is an important factor for maintaining the stability of the macromolecule, the IL chemical structure influence and the existence of specific interactions occurring between IL and DNA should not be dismissed, as shown below.

### DNA-IL Interaction Studies

Considering the set of results presented before, it is still required a deeper analysis of the molecular mechanisms and interactions occurring between ILs and DNA. Only with this information it will be possible a proper design of IL-based media for the long-term preservation of nucleic acids. In this line, UV-Vis absorption measurements were carried out to investigate interaction patterns between DNA and ILs.

**FIGURE 5:**
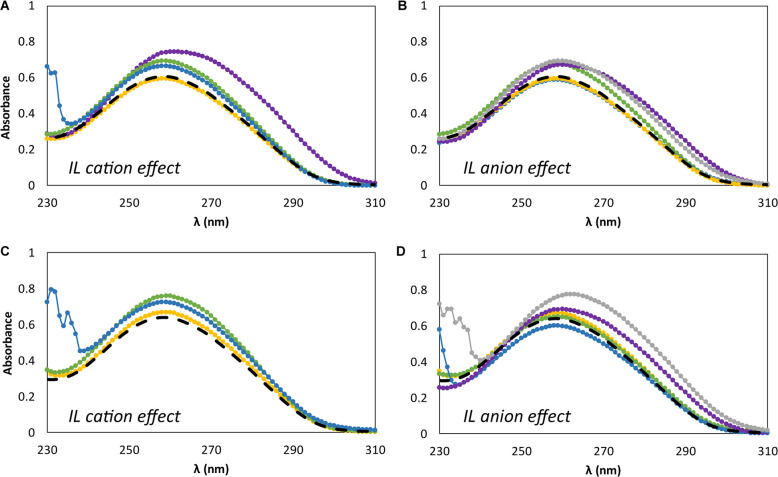
Absorption spectra of β-DNA (from salmon testes) as a function of wavelength, λ, in 10 mM of Tris–HCl buffer (pH ≈ 7.2) aqueous solutions at different concentrations of IL. **(A)** 5 wt% of bromide-based ILs: (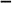) only buffer; (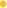) [N_111(2OH)_]Br; (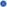) [C_2_C_1_im]Br; (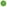) [N_4444_]Br; (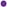) [P_4444_]Br; **(B)** 5 wt% of cholinium-based ILs. (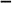) only buffer; (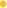) [N_111(2OH)_]Br; (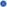) [N_111(2OH)_][Ac]; (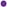) non-buffered [N_111(2OH)_][DHP]; (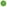) [N_111(2OH)_]Cl; (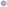) [N_111(2OH)_][Gly]; **(C)** 30 wt% of bromide-based ILs: (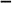) only buffer; (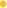) [N_111(2OH)_]Br; (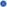) [C_2_C_1_im]Br; (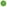) [N_4444_]Br; **(D)** 30 wt% of cholinium-based ILs. (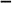) only buffer; (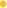) [N_111(2OH)_]Br; (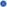) [N_111(2OH)_][Ac]; (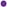) non-buffered [N_111(2OH)_][DHP]; (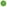) [N_111(2OH)_]Cl; (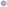) [N_111(2OH)_][Gly].

Figure 5 shows the effect of adding different concentrations of IL, namely at the minimum and maximum concentrations used in the previous studies, i.e., 5 and 30 wt%, on the dsDNA absorption spectra. Figures 5A,C represent the IL cation effect, whereas Figures 5B,D represent the IL anion effect. It should be noted that changes in position (shift) and absorbance maximum of dsDNA is associated to DNA-IL interactions and/or DNA gains and losses of stability (Haque et al., 2017; Sahoo et al., 2018). For all the absorption spectra acquired an hyperchromic effect is observed, where the absorption of dsDNA in aqueous solutions of ILs is higher than that for dsDNA in 10 mM of Tris–HCl buffer (pH ≈ 7.2). In what concerns the IL cation effect (Figures 5A,C), a specific trend of the cation structure is observed independently of the IL concentration. At 260 nm, and at the two concentrations of ILs investigated, the hyperchromicity increases in the following order: [N_111(2OH)_]Br < [C_2_C_1_im]Br ≈ [N_4444_]Br < [P_4444_]Br. An increase of the dsDNA absorption spectrum is due to the unstacking of nucleobases, as a consequence of uncoiling or denaturation processes of dsDNA, which seems to be affected by the IL chemical structure. Overall, and among the bromide-based ILs investigated, [P_4444_]Br is the IL that leads to a higher unstacking of the DNA bases, whereas [N_111(2OH)_]Br is the most promising IL to promote the DNA stability. In what concerns the IL anion effect (Figures 5B,D), a specific trend associated to the anion structure is not observed considering its impact on the dsDNA structure. Different patterns are observed when changing the IL concentration. However, amongst the cholinium-based ILs investigated, [N_111(2OH)_][Gly] is the IL that leads to a higher unstacking of nucleobases in DNA, as confirmed by the higher hyperchromic effect observed with this IL. On the other hand, [N_111(2OH)_]Br and [N_111(2OH)_][Ac] seem to be the most appropriate ILs to keep the DNA structure at the two concentrations investigated.

**FIGURE 6:**
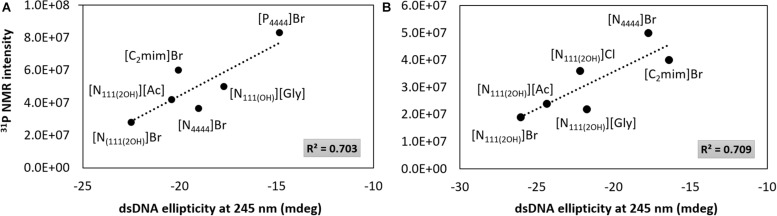
^31^P NMR intensity peaks of DNA as a function of dsDNA ellipticity at 245 nm. **(A)** IL at 5 wt%; **(B)** IL at 30 wt%.

Although the UV absorption results demonstrate that the IL influences the dsDNA helical structure, a quantitative approach was additionally carried out by ^31^P NMR analysis of the DNA phosphate backbone with aqueous solutions of ILs at 5 and 30 wt%. The intensity values of the ^31^P NMR peaks express the DNA phosphate backbone and the exposition of phosphate groups in a given environment. Figure 6 depicts the ^31^P NMR intensity values of DNA phosphate groups, in aqueous solutions of ILs at 5 and 30 wt%, as a function of dsDNA ellipticity at 245 nm that represents the helicity, whose values were taken from the data given in Figures 2, 3. Regardless the IL concentration, there is a correlation between the helicity and the ^31^P NMR intensity peaks of DNA. The higher the ellipticity of dsDNA, the higher the phosphorous peaks intensity of phosphate groups of DNA. According to Figure 6, [N_111(2OH)_]Br presents the lowest value of ellipticity and the lowest intensity peak of DNA phosphate groups, supporting the possibility of the IL cation being more strongly interacting with the phosphate backbone of dsDNA, thus lowering the phosphorous exposition to the aqueous environment. These results reinforce the higher capability of [N_111(2OH)_]Br to better stabilize dsDNA in aqueous solutions, being in agreement with the CD and UV absorption analysis (Figures 2, 3, 5). At the IL concentration of 5 wt% (Figure 6A), [P_4444_]Br presents the highest values of ellipticity and ^31^P NMR intensity associated to the phosphate groups of DNA. With [P_4444_]Br, the dsDNA macromolecule surface is more exposed to the aqueous environment and the IL cation is not preferentially interacting with the phosphate groups of the biopolymer. Accordingly, the perturbation on the dsDNA conformation due to [P_4444_]Br are not due to electrostatic interactions, but yet by dispersive interactions that may be established between the alkyl side chains of the IL cation and the nucleobases. However, with this ILs, it should be remarked that the pH effect cannot be discarded. Since [P_4444_]^+^ is also detected in ^31^P NMR spectra, an example of the obtained spectrum for the samples composed of dsDNA in 5 wt% of [P_4444_]Br in 10 mM of Tris–HCl buffer (pH ≈ 7.2) is provided in the Supplementary Figure 3, allowing to demonstrate that ^31^P NMR peaks values of DNA phosphate groups are distinguished from [P_4444_]^+^ peaks. At higher concentrations of IL (Figure 6B), i.e., at 30 wt%, there is a general trend of the cholinium-based ILs to display lower ^31^P NMR peaks intensity, reinforcing that the cholinium cation preferentially interacts with the DNA phosphate groups when compared with imidazolium and tetrabutylphosphonium/ammonium cations.

**FIGURE 7:**
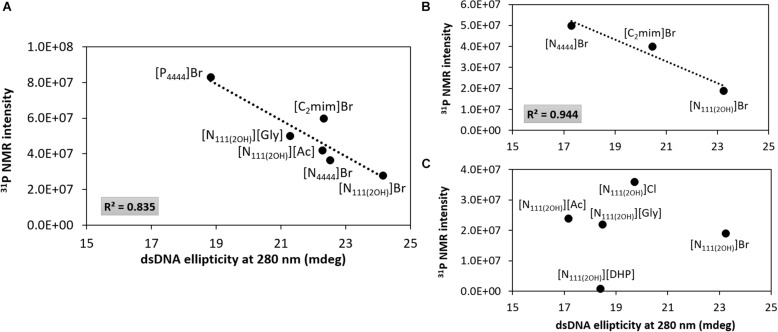
^31^P NMR intensity peak of DNA as a function of dsDNA ellipticity at 280 nm. **(A)** IL at 5 wt%; **(B)** bromide-based ILs at 30 wt%; **(C)** cholinium-based ILs at 30 wt%.

Figure 7 depicts the ^31^P NMR intensity values of DNA phosphate groups (in aqueous solutions of ILs at 5 and 30 wt%) as a function of the dsDNA ellipticity at 280 nm, representing π–π base stacking, whose values were taken from the data provided in Figures 2, 3. As dsDNA ellipticity at 280 nm represents the binding strength occurring between bases, the higher this value is the more consistent the base stacking, thus fostering a more stable double strand conformation of dsDNA. Overall, at 5 wt% of IL (Figure 7A), the ^31^P NMR peak intensity values decrease with the increase in the dsDNA ellipticity at 280 nm. This trend supports that favorable interactions of the IL cation with the DNA phosphate groups, expressed by a decrease in the NMR peak intensity, improve the DNA π–π base stacking. At 5 wt% of IL, [N_111(2OH)_]Br induces the highest dsDNA ellipticity value at 280 nm and the lowest NMR intensity peak of DNA phosphate groups, meaning that π–π base stacking occurring in dsDNA molecules are better preserved and that interactions of the IL cation with the DNA phosphate groups are preferentially established. This trend reinforces the preferential interactions of cholinium with the DNA phosphate backbone. On the other hand, π–π base stacking seems to be more relaxed in presence of [P_4444_]Br, being this the IL with the lowest ability to preserve the DNA structure as discussed above. However, at 30 wt% of IL (Figures 7B,C), no correlation between the ^31^P NMR intensity values of DNA phosphate groups as a function of the dsDNA ellipticity at 280 nm was found. These results support that at higher concentrations of ILs the molecular-level phenomenon is more complex and that other interactions different from electrostatic may play a role in the stabilization of DNA.

Overall, taking into account the data shown in Figures 6, 7, the main molecular-level mechanisms occurring in aqueous solutions containing ILs and dsDNA can be summarized as follows: (i) electrostatic interactions – at lower IL concentrations higher ^31^P NMR intensity peak values are observed, meaning that the DNA phosphate backbone is more exposed, and thus less electrostatic interactions are established between the IL cation and the DNA phosphate groups, which may be due to difficulties in “breaking” the DNA hydration shell. At higher IL concentrations, IL cations compete stronger with water molecules for the dsDNA phosphate backbone, thus decreasing the ^31^P NMR intensity peak values; (ii) dispersive interactions – the hydrophobicity of the IL cation plays a role since an increase in the DNA ellipticity and a decrease in π–π base stacking is observed with more hydrophobic ILs cations, therefore causing perturbation on the dsDNA native conformation; and (iii) the IL anion has a weaker impact when compared to the IL cation effect on interacting with DNA molecules since no correlation was found at higher concentrations of IL (Figure 7C).

The hypothesis previously addressed is in accordance with previously published results. Ding et al. (2010) demonstrated that at IL concentrations lower than 1.05 wt%, the IL cation is localized at several angstroms of distance from DNA phosphate strand, while the IL hydrophobic chains are in parallel arrangement to the DNA molecule surface. On the other hand, at higher IL concentrations, the IL cation is near to the DNA phosphate strand and the IL hydrocarbon chains are perpendicularly attached to the DNA molecule surface. Sahoo et al. (2018) described that when using cholinium-based ILs with anions derived from amino acids, the cholinium cation interacts with DNA through with an independent effect of the anions. According to the experimental and theoretical data gathered by the authors, they suggested a heterogeneity in binding modes of IL to DNA, where electrostatic interactions and H-bonding with the phosphate groups occur, while binding modes in the minor groove of dsDNA were predominantly stabilized by van der Waals interactions. Pabbathi and Samanta (2015) demonstrated that the morpholinium cation binds to the minor grooves of dsDNA and its binding is weaker when compared with the imidazolium cation. Singh et al. (2012) suggested not only the presence of electrostatic interactions between DNA and IL by fluorescence intensity measurements, but also that non-electrostatic interactions between the IL cation alkyl chain and the dsDNA base pairs are relevant. Overall, the data previously published by different authors together with the results presented in this manuscript correspond to relevant fundamental insights on the ILs effects toward DNA in aqueous solution. The gathered data are of high relevance for the preparation of effective DNA preservation media and for the design of IL-based separation processes from biological media.

## Conclusion

In this work, a screening of several ILs with different cations and anions combinations, and at different concentrations and pH values, was carried out for a deeper understanding on the molecular features responsible for the dsDNA stability and structural conformation in aqueous solutions. CD, UV absorption, and ^31^P NMR spectroscopic studies were performed, where it was observed that the pH, type, and IL concentration contribute to changes in the dsDNA conformational structure. It was observed that higher IL concentrations and hydrophobicity of the corresponding cation lead to perturbations on the structural conformation of dsDNA. Overall, the best IL identified to preserve the stability of dsDNA was [N_111(2OH)_]Br.

The obtained results allowed us to identify main interactions and phenomena responsible for the dsDNA stability in IL aqueous solutions. At lower IL concentrations, less electrostatic interactions are established between the IL cation and the DNA phosphate groups, which may be due to difficulties in “breaking” the dsDNA hydration shell. On the other hand, at higher IL concentrations, IL cations compete stronger with water molecules for the dsDNA phosphate backbone, as verified with the decrease in the ^31^P NMR intensity peak values. The hydrophobicity of the IL cation plays a main role since an increase in the DNA ellipticity and a decrease in π–π base stacking is observed with more hydrophobic ILs cations, leading to the perturbation of the dsDNA native conformation. Finally, the IL anion has a weaker impact when compared to the IL cation effect on interacting with DNA molecules. In summary, the results presented in this work provide fundamental knowledge for the application of adequate ILs in the preservation and separation and purification of nucleic acids, with significant impact in the biotechnology field.

## Data Availability Statement

The raw data supporting the conclusions of this article will be made available by the authors, without undue reservation.

## Author Contributions

TD, MF, and FS conceived and planned the work. TD carried out the experimental assays, analyzed the data, and prepared the first draft of the manuscript. All authors contributed to the interpretation and discussion of the acquired data and to the manuscript preparation.

## Conflict of Interest

The authors declare that the research was conducted in the absence of any commercial or financial relationships that could be construed as a potential conflict of interest.
