# Supplementary material for: Insights on the DNA Stability in Aqueous Solutions of Ionic Liquids
Source: Front Bioeng Biotechnol. 2020 Oct 14;8:547857. doi: 10.3389/fbioe.2020.547857 (PMC7591794; doi:10.3389/fbioe.2020.547857)
Supplement: Supplementary file 1 [file Presentation_1.pdf]

Insights on the DNA stability in aqueous solutions of ionic liquids

Teresa B. V. Dinis[1], Fani Sousa[2*] and Mara G. Freire[1*]

Supplementary Material

[1]CICECO – Aveiro Institute of Materials, Department of Chemistry, University of Aveiro, Aveiro, Portugal

[2]CICS-UBI – Health Sciences Research Center, Universidade da Beira Interior, Covilhã, Portugal

*Correspondence:

Dr. Fani Sousa

fani.sousa@fcsaude.ubi.pt

Dr. Mara G. Freire

maragfreire@ua.pt

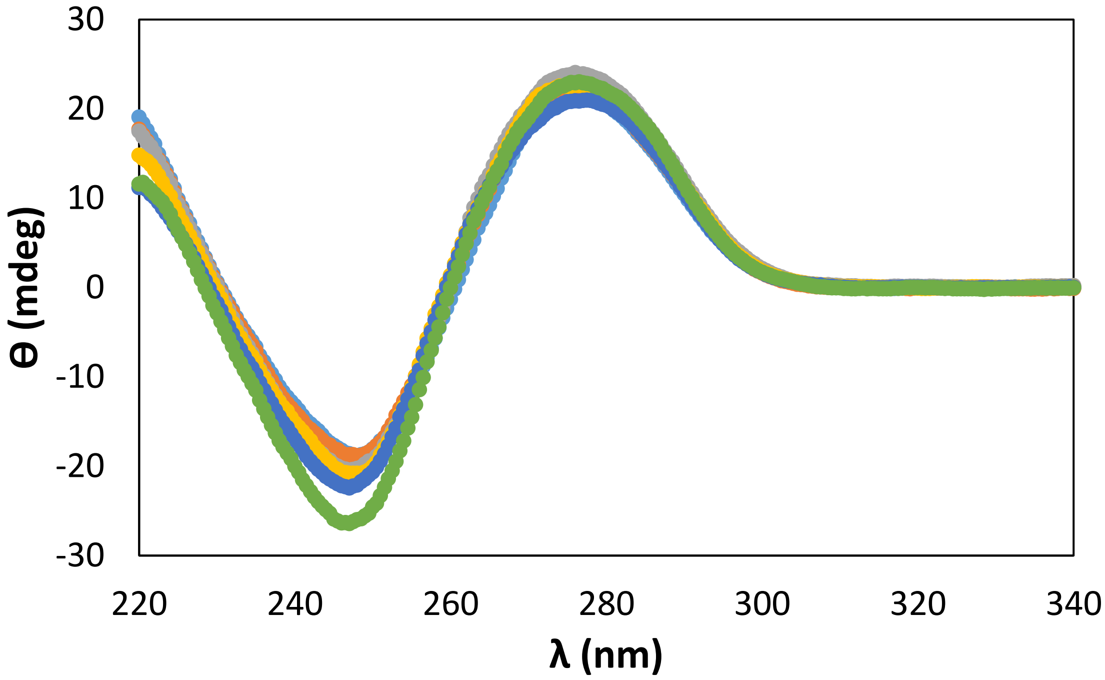

Figure S1. Circular dichroism (CD) spectra regarding the ellipticity, θ, of 0.01 g·dm^{-3} of β-DNA (from salmon testes) as a function of wavelength, λ, at different concentrations of Tris·HCl buffer at pH≈7.2: (•) 10 mM; (•) 25 mM; (•) 50 mM; (•) 100 mM; (•) 500 mM; (•) 1000 mM.

Table S1. Experimental pH values of the ILs aqueous solutions. N.D. – Non-defined value.

IL	[IL] (wt%)	pH (± 0.02)	IL	[IL] (wt%)	pH (± 0.02)
	5	7.10		5	6.13
$[N_{4444}]Br$	15	6.86	$[N_{111(2OH)}][Ac]$	15	6.23
	30	6.65		30	6.55
	5	7.00		5	3.22
$[C_2C_1im]Br$	15	5.96	$[N_{111(2OH)}][DHP]$	15	3.31
	30	4.49		30	3.60
	5	4.42		5	6.37
$[P_{4444}]Br$	15	2.15	$[N_{111(2OH)}][DHP]$ (buffered)	15	6.40
	30	N.D.		30	6.68
	5	7.14		5	5.13
$[N_{111(2OH)}]Br$	15	7.06	$[N_{111(2OH)}][Gly]$	15	5.14
	30	7.00		30	5.31
	5	7.27			
$[N_{111(2OH)}]Cl$	15	7.22			
	30	7.20			

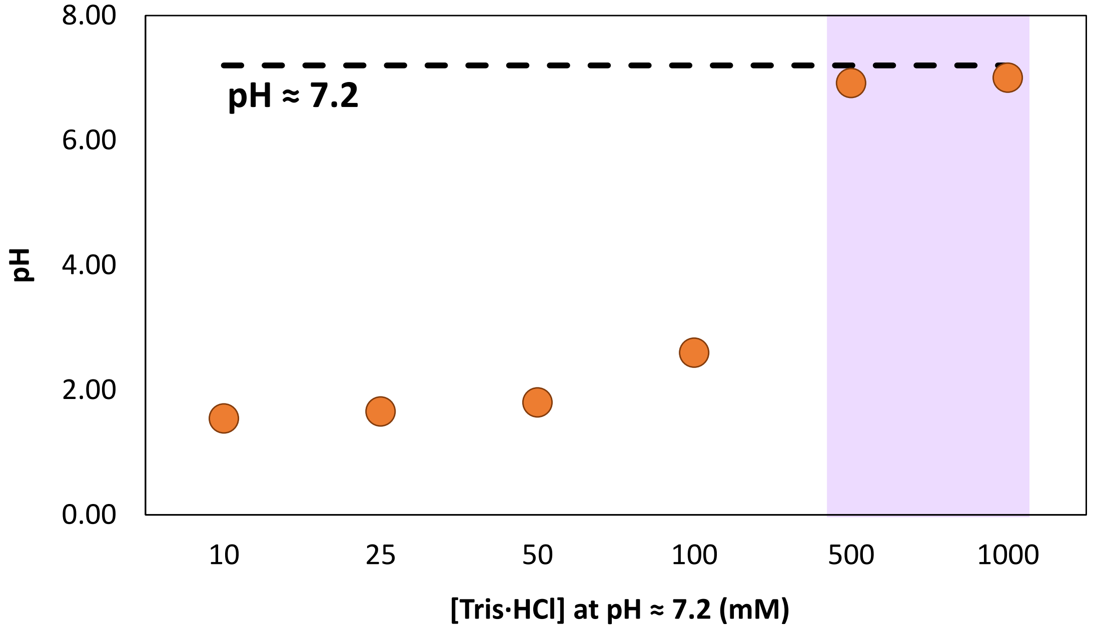

Figure S2. Effect of Tris·HCl buffer concentration, at pH≈7.2, on the pH adjustment of the aqueous solutions composed of 30 wt% of [P_{4444}]Br.

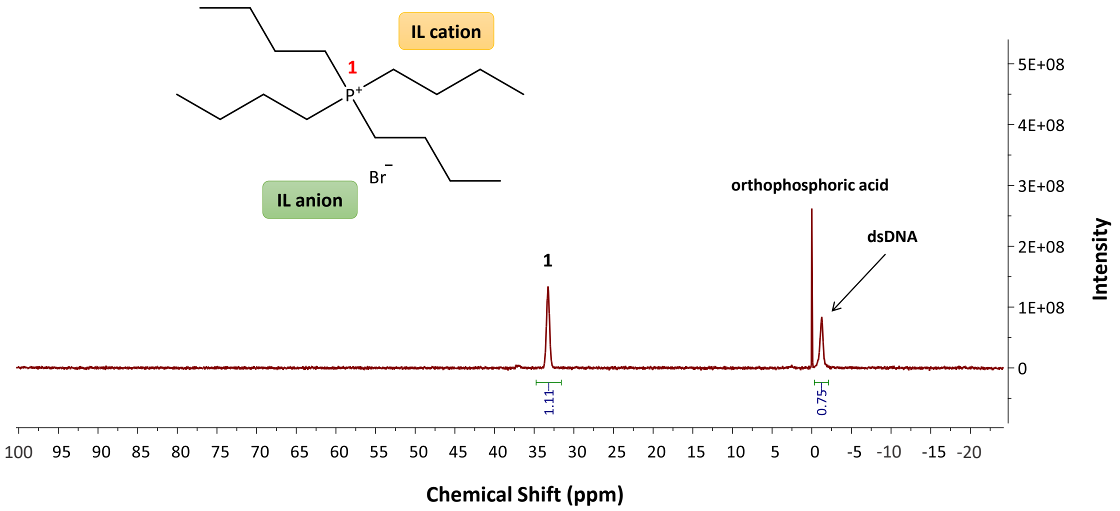

Figure S3. ^{31}P nuclear magnetic resonance (NMR) spectrum of 35 g·dm^{-3} of β-DNA in 10 mM of Tris·HCl (pH≈7.2) and 5 wt% [P_{4444}]Br in deuterium oxide (D_2O) as solvent containing trimethylsilyl propanoic acid (TSP).
